# Characterizing eating behavioral phenotypes in mood disorders: A cross-sectional investigation

**DOI:** 10.1017/S1092852926101230

**Published:** 2026-09-30

**Authors:** Elena Koning, Jill A. Jacobson, Elisa Brietzke

**Affiliations:** 1 Department of Psychiatry, Queen’s University, Canada; 2 Department of Psychology, Queen’s University, Canada

**Keywords:** Major depressive disorder, bipolar disorder, eating behavior, nutrition, mood

## Abstract

**Background:**

Maladaptive eating behaviors in mood disorders are diverse and clinically relevant but have only been characterized into phenotypes based on theoretical evidence. The objective of the current study is to assess the prevalence of maladaptive eating behavior phenotypes (EBP) in mood disorders based on empirical evidence.

**Methods:**

An online survey package including five questionnaires was administered to assess the prevalence and proportion of maladaptive eating behaviors in a mood disorder population (*n* = 100). Principal component analysis (PCA) and mixed measures analysis of variance (mANOVA) were conducted to characterize maladaptive EBPs.

**Results:**

Numerous maladaptive eating behaviors were identified in MDD and BD, including poor dietary quality, emotional eating, binge eating, and night eating behaviors. Differences between diagnostic groups are discussed. Three EBPs were characterized according to the PCA: (1) food impulsivity; (2) emotional eating; and (3) mindfulness in eating, with a significant influence of age and medication on the characterizations according to the mANOVA.

**Conclusions:**

Maladaptive eating behaviors occur in mood disorders and can be characterized into distinct EBPS. These findings should be interpreted as preliminary and hypothesis-generating given the sample size, absence of a healthy comparator group, and potential confounds. As psychiatry moves toward a more comprehensive, personalized approach to diagnosis and treatment, EBPs require prospective validation in larger, controlled investigations to determine clinical relevance.

## Introduction

Maladaptive eating behaviors are routinely observed in individuals with mood disorders, including Major Depressive Disorder (MDD) and Bipolar Disorders (BD), and impact symptom severity and the prevalence of general medical comorbidities such as obesity, diabetes, and metabolic syndrome.^1^ ^,^^2^ The heterogeneity of maladaptive eating behaviors is widespread, with some patients experiencing overeating and weight gain while others exhibit undereating and severe weight loss.^3^ Other maladaptive eating behaviors commonly observed in this population include binge eating, night eating, food addiction, and irregular meal timing.^4^ ^-^^8^

The diverse range of maladaptive eating behaviors in mood disorders was recently characterized by our group into eating behavioral phenotypes (EBPs), based on current evidence in the literature.^9^ These characterizations included high appetite, low appetite, emotional eating, food addiction, unhealthy diet, and circadian desynchronized phenotypes of eating behavior. To our knowledge, there has yet to be an investigation assessing EBPs in a real-world mood disorder population. Therefore, it is important to assess the clinical relevance and validity of these phenotypes.

The current investigation has the objective of determining phenotypes of eating behavior in mood disorders through an online survey package. The main outcome of this study will be the prevalence and proportion of each phenotype in a mood disorder population. The secondary outcome will be the characterization of meaningful phenotypic classifications of eating behaviors based on principal component analysis (PCA) of the questionnaire results.

## Methodology

### Participants

A sample of 100 individuals with a diagnosis of MDD (*n* = 50) or BD (*n* = 50) were selected for the study. Participants were recruited via numerous Ontario mood disorder support group newsletters, the Centre for Neuroscience Studies and NeuroMood Laboratory social media platforms, posters displayed in Providence Care Hospital and Kingston General Hospital, as well as the Adult Mental Health Program waitlist at Hotel Dieu Hospital in Kingston, Ontario, Canada. Inclusion criteria included: (1) age between 18 and 60 years old; (2) being clinically followed by a psychiatrist or a family doctor; (3) no changes in medication in the past 4 weeks; (7) having access to email and the internet; (8) fluent in English. Exclusion criteria included: (1) active medical comorbidity that impacts appetite (2) currently using or planning to begin using medication with the aim of losing weight. All study procedures and materials were approved by Queen’s University Health Sciences and Affiliated Teaching Hospitals Research Ethics Board.

### Instruments

Five questionnaires were selected based on the previously characterized EBPs. They evaluate appetite, emotional eating, dietary quality, circadian desynchronized eating, and food addiction behaviors.

#### Binge Eating Scale (BES)

BES is a 16-item multiple-choice questionnaire with questions based on the presence of behavioral, emotional, cognitive, guilt, and shame indicators of binge eating behavior. Each question has three or four options that are each assigned a numerical value. Scores range from 0–46 with scores 0–17 indicating little or no binge eating behavior, 18–26 indicating moderate binge eating behavior, and 27–46 indicating severe binge eating behavior. The BES has shown good reliability and sound psychometric properties upon previous assessment.^10^ ^,^^11^

#### Adult Eating Behavior Questionnaire (AEBQ)

AEBQ is a 35-item questionnaire that assesses appetitive traits, including food approach (hunger, food responsiveness, enjoyment of food, emotional overeating) and food avoidant (emotional under-eating, food fussiness, slowness in eating, satiety responsiveness) behaviors. Each item provides the option to respond strongly disagree, disagree, neither agree nor disagree, agree, or strongly agree, and scores range from 1–5 for each criterion.^12^

#### Short-Form Food Frequency Questionnaire (SFFFQ)

*SFFFQ* is a 20-item questionnaire designed to assess the consumption of certain foods over a ‘typical week’ in which respondents select one frequency option ranging from ‘rarely or never’ to ‘5+ times per day.’ The resultant diet quality score (DQS) is a calculation of fruit, vegetables, oily fish, fat and non-milk extrinsic sugar (NMES) consumption which reflects five dietary components recognized as indicators of a healthy diet.^13^ ^,^^14^ Scores of 1–3 were allocated to each component, with a score of 3 corresponding to meeting the UK dietary recommendations for that group (making the DQS range from a minimum of 5 to a maximum of 15). A healthy diet can be defined as having an overall DQS of >12. The SFFFQ also includes some basic questions about exercise and substance use.

#### Night Eating Diagnostic Questionnaire (NEDQ)

*NEDQ* is a 20-item questionnaire that can be used to establish a diagnosis of circadian desynchronized eating behavior using the proposed DSM criteria.^15^ Although it does not provide a global score of symptom severity, a hierarchical scoring method has been developed to assess mild, moderate, and full-syndrome night eaters.^16^

#### Modified Yale Food Addiction Scale (mYFAS)

mYFAS 2.0 is an abbreviated, 13-item version of the Yale Food Addiction Scale 2.0 (YFAS 2.0). The mYFAS 2.0 has one question to assess each of the 11 DSM-5 diagnostic criteria for substance-use disorders, plus 2 questions to assess clinically significant distress and impairment. ^17^ The mYFAS 2.0 has been previously shown to perform similarly on psychometric indicators as the full version of the scale and was used to assess the presence of food addiction in the current investigation.

### Procedures

As this study was designed and implemented during the COVID-19 pandemic, all study procedures were conducted completely online. Recruitment began in June 2022 and ran until July 2023. All participants completed the survey study in an asynchronous manner.

The study advertisements instructed prospective participants to follow a Google Form link that provided information about the study procedures, compensation, and time commitment. If interested in continuing with the study, participants were instructed to report their email address and diagnosis in the Google form. Within 24 hours, a member of the research team contacted participants via email and sent the letter of information/consent (LOIC) and screening/sociodemographic forms through a secure Queen’s University OneDrive link. The LOIC explained the nature and rationale for the survey study and provided participants with a clear description of the study procedures. The LOIC also described risks and benefits of participating in the study, withdrawal options, confidentiality, and compensation. Participants were instructed to read the letter of information carefully and, if they consented to participate, complete and return the forms to the research team through a distinct, secure OneDrive link.

Based on inspection of the screening/sociodemographic form, a member of the research team assigned eligible participants a unique identifier. All the unique participant identifier names, and email addresses were kept in a secure OneDrive Folder (a separate Folder from all other data related to the survey study). Eligible participants were sent the questionnaire package through another secure OneDrive link and instructed to include their unique participant identifier and date at the top of each questionnaire. Participants were also instructed to complete the questionnaires at their own convenience and to answer all questions as accurately as possible.

### Data analysis

The questionnaires were scored by the research team and organized into an Excel file on the Queen’s University OneDrive Folder along with the sociodemographic information. Upon completion of the recruitment period, all data were cleaned and transferred to RStudio® software (version 2023.06.2 + 56, Posit Software, PBC) for analysis.^18^ The prevalence and proportion of maladaptive eating behaviors were calculated and presented as descriptive statistics. Analysis of variance (ANOVA) was conducted to identify significant differences in eating behaviors between the groups. Following, a 2-tailed Pearson’s correlation was used to identify any significant associations between maladaptive eating behaviors in the total mood disorder population and principal component analysis using the psych package was conducted to identify phenotypic classifications.^19^ An oblique rotation using the GPA rotation package was used to make the solution interpretable.^20^ A mixed analysis of variance was used to determine whether age, gender, BMI, medication, or lifestyle factors (exercise, alcohol use, cannabis use, smoking) were significantly related to the identified components.

## Results

### Sample characteristics

The characteristics of the study sample are shown in Table 1. Half of the study sample included individuals with MDD (*n* = 50), followed by BDII (*n =* 31) and BDI (*n* = 19). Most respondents in each group were female (68% MDD, 63.2% BDI, 58.1% BDII) and identified their ethnicity as Caucasian (46% MDD, 78.95 BDI, 71% BDII). Regarding dietary content and quality scores (Table 1), NMES consumption varied significantly between the diagnostic groups (*F* (2, 97) = 5.089, *p* = 0.008), whereas dietary composition of fruit, vegetables, oily fish and fat, as well as total DQS, were not significantly different between groups. According to the SFFFQ, 80% of individuals with MDD and 100% of individuals with BD did not meet dietary guidelines for a healthy diet (Table 1).

**Table 1.** Sociodemographic Characteristics and Dietary Data of the Sample Table 1. Long description.

Sociodemographic characteristics			
	MDD, *N* (%)	BDI, *N* (%)	BDII, *N* (%)
Participants	50	19	31
Age (years) Less than 20 20–29 30–39 40–49 50–59	5 (10) 28 (56) 13 (26) 3 (6) 1 (2)	1 (5.3) 8 (42.1) 2 (10.5) 7 (36.8) 1 (5.3)	1 (3.2) 10 (32.3) 14 (45.2) 5 (16.1) 1 (3.2)
Gender Male/Female/NB	16 (32)/ 34 (68)/ 0(0)	7 (36.8)/ 12 (63.2)/ 0(0)	11 (35.5)/ 18 (58.1)/ 2 (6.5)
Employment status Full time/Part time/Other	26 (52)/ 13 (36)/ 11 (22)	9 (47.4)/ 8 (42.1)/ 2 (10.5)	14 (45.2)/ 14 (45.2)/ 3 (9.7)
Highest education High school diploma Some college, no diploma Community college Diploma Bachelor’s degree Master’s degree Doctoral degree	6 (12) 8 (16) 6 (12) 28 (56) 2 (4)	5 (26.3) 4 (21.1) 5 (26.3) 4 (21.1) 1 (5.3)	12 (38.7) 8 (25.8) 9 (29) 2 (6.5)
Income Less than $15 000 $15 000–$24 999 $25 000–$49 999 $50 000 and over Prefer not to answer	15 (30) 10 (20) 7 (14) 15 (30) 3 (6)	1 (5.3) 8 (42.1) 6 (31.6) 4 (21.1)	6 (19.4) 10 (32.3) 13 (41.9) 2 (6.50 2 (6.5)
Ethnicity South Asian Southeast Asian East Asian Caucasian Middle Eastern African American Other Latin-American Indigenous Prefer not to answer	2 (4) 6 (12) 2 (4) 23 (46) 1 (2) 10 (20) 1 (2) 2 (4) 3 (6)	1 (5.3) 15 (78.9) 1 (5.3) 1 (5.3) 1 (5.3)	1 (3.2) 2 (6.5) 22 (71) 4 (12.9) 1 (3.2) 1 (3.2)
BMI <18.5 18.5–24.9 25–29.9 ≥ 30	5 (10) 21 (42) 12 (24) 12 (24)	16 (84.2) 3 (15.8)	5 (16.1) 17 (54.8) 5 (16.1) 4 (12.9)
Medications Antidepressant Mood stabilizer Anticonvulsant Antipsychotic None	26 (52) 1 (2) 23 (46)	1 (5.3) 11 (57.9) 7 (36.8) 1 (5.3)	4 (12.9) 16 (51.6) 7 (22.6) 4 (12.9) 2 (6.5)
Alcohol (Drinks per Week) I rarely/never drink alcohol Less than 14 units Between 14 & 21 units More than 21 units	33 (66) 14 (28) 2 (4) 1 (2)	8 (42.1) 7 (36.8) 4 (21.1)	11 (35.5) 16 (51.6) 4 (12.9)
Smoking Never smoked >100 cigarettes Current smoker Ex-smoker	36 (72) 5 (10) 9 (18)	10 (52.6) 6 (31.6) 3 (15.8)	10 (32.3) 15 (48.4) 6 (19.4)
Cannabis (Routine use) Yes/ No	3 (6)/ 47 (94)	0 (0)/19 (100)	1 (3.2)/ 30 (96.8)
Moderate or Vigorous Exercise last week < 150 min/ ≥ 150 min	37 (74)/ 13 (26)	16 (84.2)/ 3 (15.8)	29 (93.5)/ 2 (6.5)

Dietary content and quality			
	MDD^a^	BDI^a^	BDII^a^
DQS	9.80 (1.895)	10.53 (1.467)	10.48 (1.180)
Fruit	1.84 (.738)	2.00 (.00)	1.87 (.341)
Vegetables	1.82 (.825)	1.89 (.567)	1.84 (.523)
Oily Fish	2.00 (.728)	1.74 (.562)	1.74 (.575)
Fat	1.92 (.900)	2.16 (.834)	2.32 (.748)
NMES	2.22 (.840)	2.74 (.562)	2.65 (.608)

Respondents not meeting dietary guidelines for a healthy diet			
	MDD^b^	BDI^b^	BDII^b^
Fruit	40 (80)	19 (100)	31 (100)
Vegetables	37 (74)	17 (89.5)	29 (93.6)
Oily Fish	37 (74)	18 (94.7)	29 (93.5)
Fat	32 (64)	11 (57.9)	16 (51.6)
NMES	26 (52)	4 (21.1)	9 (29)
DQS	46 (92)	18 (94.7)	31 (100)

Abbreviations: NB, non-binary; min, minutes.

a Values presented as mean (standard deviation), unless otherwise specified.

b Values presented as counts (percentages), unless otherwise specified.

### Prevalence of maladaptive eating behaviors in mood disorders

Numerous maladaptive eating behaviors were identified across diagnostic groups. Each diagnostic group displayed binge eating behavior to some degree, with most participants with MDD (60%) and BDII (58.1%) displaying moderate or severe binge eating behavior. For participants with BDI, the majority (57.9%) scored in the “Little or no binge eating behavior” category (Table 2). The diagnostic groups did not differ significantly in binge eating behavior (*F* (2, 97) = 0.510, *p* = 0.602).

**Table 2.** Maladaptive Eating Behaviors Table 2. Long description.

Binge eating behavior			
	MDD	BDI^a^	BDII^a^
Little or no binge eating behavior Moderate binge eating behavior Severe binge eating behavior	20 (40) 18 (36) 12 (24)	11 (57.9) 4 (21.1) 4 (21.1)	13 (41.9) 10 (32.3) 8 (25.8)

Emotional eating			
	MDD	BDI^a^	BDII^a^
Enjoyment of Food	4.13 (.83)	4.32 (.77)	4.29 (.95)
Emotional overeating	2.87 (1.06)	3.73 (.76)	3.09 (1.18)
Emotional under-eating	3.07 (1.03)	2.43 (.52)	2.53 (1.06)
Food fussiness	2.36 (1)	2.27 (.84)	2.34 (1.14)
Food responsiveness	3.58 (.89)	3.91 (.54)	3.53 (.87)
Slowness in eating	3.02 (1.03)	2.93 (.98)	2.86 (.99)
Hunger	3.47 (.73)	3.11 (.92)	3.11 (.78)
Satiety responsiveness	2.62 (1.06)	1.75 (.67)	2.18 (1.12)

Night eating behavior			
	MDD	BDI^a^	BDII^a^
Normal	21 (42)	10 (52.6)	15 (48.4)
Mild night eater	6 (12)	2 (10.5)	6 (19.4)
Moderate night eater	14 (28)	3 (15.8)	3 (9.7)
Full syndrome night eater	9 (18)	4 (21.1)	7 (22.6)

Food addiction			
	MDD	BDI^a^	BDII^a^
No food addiction	33 (66)	17 (89.5)	26 (83.9)
Moderate food addiction	3 (6)	1 (5.3)	2 (6.5)
Severe food addiction	14 (28)	1 (5.3)	3 (9.7)

a Values presented as counts (percentages), unless otherwise specified.

The AEBQ revealed the presence of emotional eating behaviors in each diagnostic group, with BDI participants demonstrating the highest average score on the emotional overeating subscale. Participants with MDD demonstrated the highest average score on the emotional undereating subscale (Table 2). No significant differences in enjoyment of food, food fussiness, food responsiveness, slowness in eating, or hunger were observed between the diagnostic categories. Significant differences in emotional overeating (*F* (2, 97) = 4.620, *p* = 0.012) and emotion undereating (*F* (2, 96) = 4.400, *p* = 0.015) were identified between the diagnostic groups as well as satiety responsiveness (*F* (2, 97) = 5.339, *p* = 0.006).

Most participants with MDD (58%) and BDII (51.6%) met the criteria for night eating behavior (Table 2). In the MDD group, the proportion of participants meeting the criteria for mild, moderate, and full syndrome night eating was 12%, 28%, and 18%, respectively. Proportions of BDII in each respective category were 19.4%, 9.7%, and 22.6%. For participants with BDI, 52.6% were categorized as “normal” according to the NEDQ questionnaire, with 10.5%, 15.8%, and 21.1% meeting the criteria for mild, moderate, and full syndrome night eating, respectively. The diagnostic groups did not differ significantly in night eating behavior (*F*(2, 97) = 0.275, *p* = 0.760).

In all diagnostic groups, most participants displayed no food addiction, according to the YFAS questionnaire, although 28% of participants in the MDD group scored in the ‘severe food addiction’ category (Table 2). The difference in food addiction between groups was statistically significant (*F* (2, 97) = 3.551, *p* = 0.032).

### Correlations between maladaptive eating behaviors

The correlational analysis was conducted to explore the maladaptive eating behaviors that tend to co-occur in individuals with mood disorders. As visualized in the heat map (Supplementary Material), numerous significant associations were found between the maladaptive eating behaviors identified in the survey study, with the 0.01 significance level indicated in boldface.

### Principal component analysis of maladaptive eating behaviors

In the PCA, based on the parallel analysis and scree plot, three components, which accounted for 59% of the total variance in responses, were retained (Supplementary Material). The correlations between each component and each item are shown in Table 3.

**Table 3.** Factor Loadings for Eating Behavior Scores by Principal Component Number Table 3. Long description.

Eating behavior score	PC1	PC2	PC3
BES score	**0.65** ^a^	−0.15	−0.61^a^
DQS score	−0.04	−0.09	**0.51** ^a^
rNEDQ score	**0.73** ^a^	−0.09	0.00
YFAS score	**0.68** ^a^	−0.28	−0.37^a^
Food fussiness	0.03	**0.52** ^a^	−0.24
Food responsiveness	−**0.63** ^a^	0.62^a^	−0.06
Enjoyment of food	−0.22	**0.76** ^a^	0.05
Emotional overeating	−0.34^a^	**0.43** ^a^	0.08
Emotional undereating	−0.02	**0.75** ^a^	0.20
Slow necessitating	−0.04	0.27	**0.81** ^a^
Hunger	**0.81** ^a^	0.02	−0.03
Satiety responsiveness	−0.14	**0.83** ^a^	0.18
SS loadings	2.64	2.84	1.57
Proportion variance	0.22	0.24	0.13
Cumulative variance	0.22	0.46	0.59
Proportion explained	0.37	0.40	0.22
Cumulative proportion explained	0.37	0.78	1.00

a Factor loadings with an absolute value of >0.30, with highest loadings corresponding to the relevant component shown in boldface.

Questionnaires that correlated with a component greater than 0.30, indicated in boldface in Table 3, were the causal indicators of that component, and based on this pattern of questionnaires, each component was interpreted as a unique phenotype of maladaptive eating behaviors in the mood disorder population. The first component was named food impulsivity and included binge eating, night eating, food addiction, food responsiveness, and hunger. The second component was named emotional eating and consisted of food fussiness, enjoyment of food, emotional overeating, emotional undereating, and satiety responsiveness. The third component, labeled mindfulness in eating, had just two indicators: diet quality and slowness in eating. Individual component scores are shown in Figure 1, visualized according to the highest component score.

**Figure 1.**
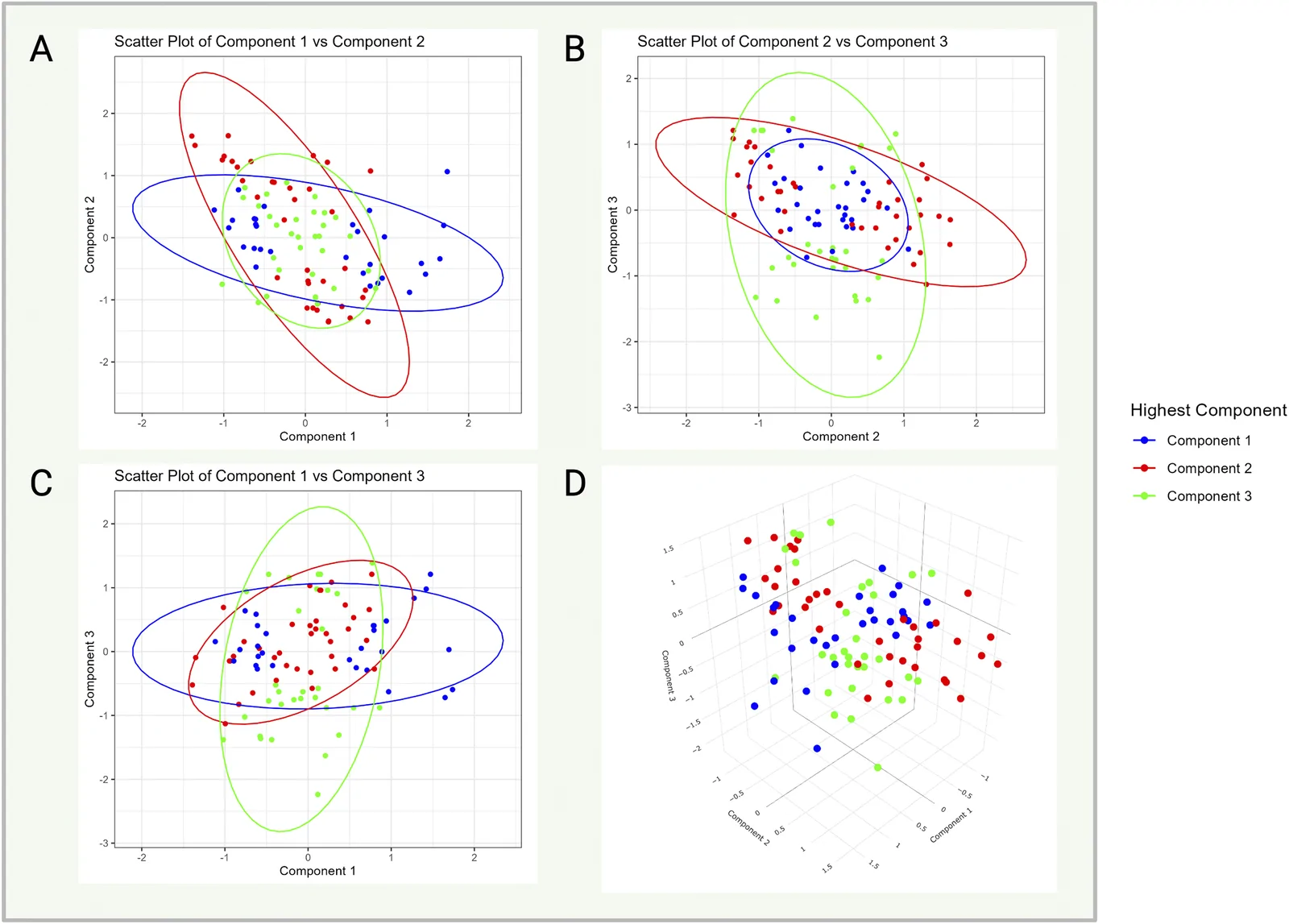
Principal components are shown in 2-dimensional plots A, B and C with colored dots representing each participants’ component scores, categorized according to the component they contribute the most variance to. Blue, red and green represent components 1 (food impulsivity), 2 (emotional eating) and 3 (mindfulness in eating), respectively. A 3-dimensional visualization of participants’ component scores is shown in D. Figure 1. long description.

### Mixed analysis of variance of factors influencing phenotypes

There is a statistically significant influence of age (*p* < 0.01) and medication use (*p* < 0.05) on the components identified in the PCA, indicated as boldface *F*-values in Table 4. No statistically significant influence of diagnosis, gender, BMI, cannabis use, alcohol use, exercise, or smoking was identified according to the mANOVA.

**Table 4.** Repeated Measures Analysis of Variance for Factors Influencing Component Scores Table 4. long description.

Factor	df	MSE	*F*-value	ges	*p*-value
Diagnosis	2, 96	0.33	2.20	.009	.116
Age	4, 94	0.31	3.75**	.031	.007
Gender	2, 96	0.34	0.56	.002	.574
Medication	4, 94	0.32	2.71*	.022	.035
BMI	67, 31	0.29	1.26	.385	.241
Cannabis use	1, 97	0.34	0.17	<.001	.683
Alcohol use	3, 95	0.34	0.56	.004	.642
Exercise	1, 97	0.34	1.45	.003	.232
Smoking	2, 96	0.34	0.88	.004	.418

Abbreviations: df, degrees of freedom; MSE, mean square; ges, generalized eta squared.

Statistical significance: **p < 0.01, *p < 0.05. Statistically significant *F*-values are indicated in boldface.

## Discussion

The current study provides evidence of maladaptive eating behaviors in mood disorders and three unique phenotypic classifications based on cross-sectional evidence from individuals with MDD and BD. Based on the study design, the findings described below should be interpreted as preliminary and hypothesis-generating.

### Maladaptive eating behaviors

Maladaptive eating behaviors identified in mood disorders include poor dietary quality, binge eating behavior, emotional eating, and night eating behavior. These results are consistent with current literature on maladaptive eating behaviors in this population, as described in previous work by our group and others.^9^ ^,^^21^ In particular, an increased preference and consumption of refined carbohydrates,^5^ ^,^^22^ ^,^^23^ including higher glycemic load ^24^ ^-^^27^ and saturated fat when compared to healthy controls (*p* < 0.001).^28^ In the current study, most individuals with mood disorders (80% in MDD and 100% in BD) did not meet the dietary guidelines for a healthy diet, consistent with this evidence.

The NMES category varied significantly between diagnostic groups, reflecting sugars such as syrups, table sugar, soft drinks, and other sweets. Individuals with MDD consumed more NMES than people with BD. Although few studies have compared NMES consumption between mood disorder diagnoses, a cross-national epidemiological study found a significant positive correlation between sugar consumption and the annual rate of depression (*r* = 0.948, *p* = 0.004).^29^

Binge eating behavior was identified in most individuals with MDD (60%) and BDII (58.1%) in the current study, whereas most participants with BDI (57.9%) displayed little or no binge eating behavior. Differences in the prevalence of binge eating behavior were not statistically significant between diagnostic groups. In a different sample of MDD and BD patients (*n* = 631), 20.6% displayed binge eating behaviors, with a higher percentage in BD (25.4%) compared with MDD.^8^ Similarly, in a study of outpatients with BDI (*n* = 51), approximately 18% of participants met the criteria for binge eating disorder. It is important to note that the current survey study assessed binge eating behavior via the BES scale, while the other study used the Binge Eating Disorder Clinical Interview to determine the presence of binge eating disorder^30^ and Woldeyohannes et al. used the DSM-5 MINI criteria. Nonetheless, numerous lines of evidence have identified binge eating behavior in individuals with mood disorders and associations with increased disease burden, suicidality, substance use, and BMI.^5^ ^,^^31^

Emotional eating is a shared characteristic between eating disorders and mood disorders and may mediate the association between conditions. In the current analysis, emotional eating was identified in all groups. Significant differences in emotional overeating and undereating were identified between diagnostic groups, with the highest scores in BDI and MDD, respectively. In a different cross-sectional analysis, Martin et al. found an increased prevalence of emotional eating behavior in individuals with BD as compared to controls.^5^ Further, emotional eating is associated with increased severity of depressive symptoms ^7^ ^,^^32^ ^-^^34^ with altered serotonergic signaling providing a potential neurobiological mechanism for this link.^35^ To our knowledge, no study has compared the prevalence of emotional eating behaviors between MDD and BD populations.

Night eating behavior was identified as prevalent to some degree in all diagnostic groups, with most participants MDD (58%) and BDII (51.6%), with 52.6% of participants in the BDI group classified as “normal.” A significant proportion of individuals in each group met the diagnostic criteria for night eating syndrome (22.6% of BDII, 21.1% of BDI, and 18% of MDD), with no significant differences between diagnostic groups. Desynchronization of circadian behaviors, including eating rhythms, is associated with more severe symptoms and metabolic comorbidities in mood disorders, with the majority of evidence focused on BD.^1^ ^,^^36^ ^-^^38^ Night eating syndrome and evening preference are more prevalent in BD than in the general population and represent a risk factor for depression, supporting the current results of the NEDQ survey.^6^ ^,^^39^ ^,^^40^

A significant difference in the prevalence of food addiction was identified between diagnostic groups. Although most participants did not display food addiction behaviors, 28% of participants in the MDD group scored in the “severe food addiction” category. Similarly, the literature to date suggests that approximately 25% of individuals with depression meet the criteria for food addiction.^7^ ^,^^41^ Increased impulsivity, reward system dysfunction, and substance use in mood disorders, the presence of food cravings, as well as the excessive consumption of highly palatable foods may be related to food addiction behaviors in this population.^9^

### Correlations between maladaptive eating behaviors

Numerous associations between eating behaviors were identified as significant in mood disorders. Our findings are largely consistent with the literature, although differences could be attributed to the small sample size and the tendency for correlations among subscales of the same questionnaire to be identified. As correlations did not easily allow for the identification of phenotypes based on eating behavior scores and positive results are susceptible to the effect of multiple comparisons, the PCA was conducted to characterize eating phenotypes in mood disorders.

### Phenotyping results

Eating behaviors were categorized into three phenotypes: food impulsivity, emotional eating, and mindfulness in eating, according to the PCA results. From these data, these phenotypes emerge as meaningful classifications of eating behavior in mood disorders.

#### Phenotype 1: Food impulsivity

Food impulsivity, the first component identified, includes behaviors of binge eating, night eating, food addiction, food responsiveness, and appetite. Both empirical and theoretical evidence provide a basis for this phenotype. For example, higher scores of impulsivity as a psychological trait are associated with all phases of mood disorders when compared to healthy controls.^42^ ^-^^46^

Impulsivity is also associated with numerous maladaptive behaviors, including binge eating, night eating, and food addiction.^47^ This is one of the reasons why substance use disorders are more common in mood disorders when compared to the general population.^48^ ^,^^49^ Food addiction could be conceptualized as a similar phenomenon in which the substance is food itself. Although neurobiological mechanisms are distinct, neuroimaging studies reveal similar mechanisms in addiction to food and other substances, including altered dopaminergic neurotransmission, emotional dysregulation and impulsivity.^50^ ^,^^51^ Among patients with binge eating disorder, substance use disorders and mood disorders tend to co-occur and are associated with a more severe illness course.^52^ ^-^^54^

Appetite and food responsiveness are also linked and may be neurobiologically related to impulsivity, as both are associated with peripheral signals, leading to an increased desire to eat. Neuroendocrine signals associated with eating behavior have been implicated in the complexity of food addiction when compared to other substance use issues,^51^ providing an explanation for the relevance of these traits to the food impulsivity phenotype. The overlap in neurobiological processes related to eating behavior, reward, and impulsivity provides a theoretical basis for these behaviors to co-occur together, as observed in the current analysis.

#### Phenotype 2: Emotional eating

The second component was interpreted as emotional eating, including food fussiness, enjoyment of food, emotional overeating, emotional undereating, and satiety responsiveness. Emotional eating is associated with the activity of brain regions involved in self-control, with differences in response to food restraint and approach, highlighting a neurobiological basis for emotional over- and under-eating.^55^ Energy balance hormones such as orexin and ghrelin influence the activity of brain regions associated with emotional overeating, including midbrain dopaminergic neurons.^56^ This may explain the relevance of satiety responsiveness to this phenotype, with increased and decreased satiety responsiveness in emotional under- and over-eating, respectively. Individuals with depression-related changes in appetite demonstrate abnormal satiety signaling.^3^ ^,^^57^ ^,^^58^

Enjoyment of food contributes to both emotional over- and under-eating. Altered pleasure responses to food are associated with reduced appetite in mood disorders, which may reflect parallel mechanisms of anhedonia in which reduced enjoyment of food, like other pleasure-inducing behaviors, reduces the likelihood of the individual engaging in them.^59^ ^,^^60^ Further, individuals with BD have demonstrated greater perceived hunger than healthy controls, which may be associated with an increased threshold of food needed to elicit a normal pleasure response from eating.^58^

#### Phenotype 3: Mindfulness in eating

The third component, mindfulness in eating, encompasses diet quality and slowness in eating. Mindful eating involves the non-judgmental awareness of physical and emotional sensations associated with food. Reduced mindful eating has been observed in individuals with mood disorders and is associated with symptom severity and quality of life.^61^ Both dietary quality and eating rate reflect important aspects of food-associated mindfulness. Monroe described eating rate as the first basic principle of mindful eating, in which eating slower allows satiety signals to effectively act on the brain, leading to satiety and the avoidance of caloric overconsumption.^62^

Neuroendocrine signals released following the digestion and absorption of food are involved in the rate at which satiety is achieved following a meal. The presence of distractions during a meal also limits mindful eating due to the reduced ability to assess internal sensory cues such as taste and feelings of satiation.^63^ ^,^^64^ It is possible that individuals who tend to display more mindfulness in the process of eating, including a slower rate and fewer distractions, tend to also extend this mindfulness to their food choices as well. This is supported by cross-sectional evidence, indicating a significant association between mindful eating and diet quality.^65^ ^-^^67^

Increased mindfulness in eating contributes to reduced caloric consumption of low-quality foods and may explain the relationship between eating behaviors associated with this phenotype.^65^ ^,^^68^ The promotion of a healthy diet and mindfulness surrounding eating represents important goals for the improvement of maladaptive eating behaviors in mood disorders. Mindfulness-based interventions have been employed for both disordered eating and mood disorders, shown to successfully reduce total caloric intake, maladaptive eating behaviors, and symptoms of mood disorders.^69^ Mindfulness-based interventions focused on eating behavior involve reducing the rate of food consumption during a meal, reducing meal portions, and eliminating distractions while eating.^62^ Mechanistically, increasing momentary awareness of hunger and satiety cues may be responsible for the beneficial effects of these interventions.^70^

### Factors influencing eating behavior phenotypes

The phenotypic classifications identified in the PCA were significantly influenced by age and medication use, according to the results of the mANOVA. Numerous lines of evidence suggest a role of age on eating behavior in which younger age is associated with worse dietary habits and eating impairment when compared with older adults.^71^ For example, in a population of 12- to 25-year-old females, younger age was found to augment the association between mood symptoms and disordered eating.^72^ ^,^^73^ Potential explanations for this trend include the influence of physician advice, social norms in different age groups, as well as changes in the gastrointestinal system and hedonic value of food, which occur alongside aging.^74^

Medications commonly prescribed for mood disorders are known to have a potential impact on eating behavior, in line with the influence of medication use on phenotypic classification in the current analysis. One of the primary concerns with the impact of psychotropic medications on eating behavior is the risk of poor appetite control, weight gain, and metabolic complications.^75^ ^-^^78^

Factors such as lifestyle, BMI, gender, and psychiatric diagnosis have also been shown to influence eating behavior, despite not being identified as having a significant effect in the mANOVA.^79^ ^,^^80^ Substance abuse is linked with disordered eating behaviors, including associations between alcohol consumption and binge eating, stimulant use and purging, as well as tobacco use and dieting and bingeing.^81^ ^-^^83^ Lifetime alcohol use is significantly higher in individuals with BD that exhibit binge eating behaviors.^8^ Cannabis is the most commonly used substance among individuals with disordered eating symptoms and is known to impact neuroendocrine signals implicated in hunger and satiety.^84^ ^,^^85^ While it is surprising that these lifestyle factors did not influence phenotypic classifications, this may be attributed to variability among participants.

Anthropometric variables did not influence the phenotypic classifications, despite evidence demonstrating that individuals with MDD and BD tend to have an increased waist circumference, higher levels of visceral adiposity, and meet the criteria for metabolic syndrome more frequently, when compared with healthy controls.^86^ ^-^^88^ Although gender was not identified to have a significant effect on the phenotypes, several studies have found gender differences in maladaptive eating behaviors, typically being more severe in women.^89^ In one study, the effect of emotional eating on mood was modulated by gender.^90^

Despite differences in the prevalence of maladaptive eating behaviors across diagnostic groups, the results of the PCA did not indicate a significant influence of diagnosis on the phenotypic classifications. This finding suggests that maladaptive eating behaviors may not be significantly different between mood disorder categories. Similarly, Zarouna et al. highlight the heterogeneity of eating dysfunction in mood disorder patients, which limits the ability to identify consistent trends at the clinical population level.^91^

Previous work by our group identified 5 eating behavioral phenotypes (EBPs) based on evidence in the literature to date, including high appetite, low appetite, emotional eating, low diet quality, food addiction, and circadian desynchronized EBPs. As this work was purely theoretical, the phenotypes identified in the current study differ.^9^ To our knowledge, no studies have characterized eating behavior phenotypes in mood disorders based on empirical evidence, limiting the opportunity to make further comparisons.

### Limitations

The results of this study should be considered in light of some limitations. First, no clinical data were collected on affective symptoms in the study population. Further, the cross-sectional nature of the study limits the ability to make causal assumptions or determine the chronicity of maladaptive eating behaviors and mood symptoms. Secondly, individuals with a diagnosis of disordered eating were not excluded from the current analysis to improve the generalizability of the results. It is also possible that some of the identified maladaptive eating behaviors represent the presence of an undiagnosed eating disorder, or a result of medication use, which significantly influenced the identified components. Third, no healthy comparator group was included. Although this study was focused on phenotyping individuals with mood disorders, the exclusion of healthy control groups limits the ability to make comparisons and gain insight about the degree to which eating behaviors are abnormal in this patient population when compared to the wider general population. Fourth, the sample size and demographic characteristics cannot represent the full mood disorder population, limiting the generalizability of the results. Furthermore, the sample size may be considered small relative to the number of variables entered into the PCA. The results of the mANOVA may not have identified an effect of certain factors due to the relatively small sample size and heterogeneity in responses across participants. The retrospective nature of the data collected in the present study should be considered in light of the limitations of human memory. Another limitation of questionnaire-based investigations is the influence of social desirability, in which participants may choose to report untruthfully in order to be viewed favorably by others.^92^ ^,^^93^

Collectively, these limitations warrant a cautious interpretation of these findings as preliminary and hypothesis-generating, rather than established clinical phenotypes. Prospective studies in larger, controlled cohorts are needed before these phenotypes can be considered validated.

## Conclusion

Taken together, maladaptive eating behaviors occur in mood disorders, including poor dietary quality, emotional eating, binge eating, and night eating behaviors. Three preliminary, hypothesis-generating phenotypes of maladaptive eating behaviors were identified in this population, including phenotypes related to food impulsivity, emotional eating, and mindfulness in eating. The influence of factors such as age and medication use highlights the importance of considering multifactorial contributions to maladaptive eating behavior classifications. Prospective studies in larger, controlled cohorts that include a healthy comparator group are needed to validate these phenotypes before they may inform clinical practice. Overall, a consideration for maladaptive eating behavior in research and clinical care for mood disorders will contribute to moving toward a more comprehensive approach to mental health assessment and treatment.

## Supporting information

10.1017/S1092852926101230.sm001 Koning et al. supplementary material Koning et al. supplementary material

## Supplementary material

The supplementary material for this article can be found at http://doi.org/10.1017/S1092852926101230.

## Long descriptions

### Table 1. Long description

The table is divided into three primary sections.

1. Sociodemographic characteristics:

- Total participants: M D D 50, B D I 19, B D I I 31.

- Age: The largest group for M D D is 20 to 29 years (56 percent), for B D I is 20 to 29 years (42.1 percent), and for B D I I is 30 to 39 years (45.2 percent).

- Gender: Females represent the majority in all groups (M D D 68 percent, B D I 63.2 percent, B D I I 58.1 percent).

- Employment: Full-time employment is highest in M D D (52 percent) and B D I (47.4 percent).

- Education: Bachelor’s degrees are most common in M D D (56 percent), while High school diplomas are most common in B D I I (38.7 percent).

- Ethnicity: Caucasian is the most frequent ethnicity across all groups (M D D 46 percent, B D I 78.9 percent, B D I I 71 percent).

- B M I: The 18.5 to 24.9 range is most common for M D D (42 percent) and B D I I (54.8 percent), while B D I has 84.2 percent in that range.

- Medications: Antidepressants are used by 52 percent of M D D, 5.3 percent of B D I, and 12.9 percent of B D I I participants.

- Lifestyle: Most participants rarely or never drink alcohol and have never smoked more than 100 cigarettes. Exercise levels below 150 minutes per week are prevalent (M D D 74 percent, B D I 84.2 percent, B D I I 93.5 percent).

2. Dietary content and quality (Mean and Standard Deviation):

- D Q S: M D D 9.80 (1.895), B D I 10.53 (1.467), B D I I 10.48 (1.180).

- Fruit: M D D 1.84, B D I 2.00, B D I I 1.87.

- Vegetables: M D D 1.82, B D I 1.89, B D I I 1.84.

- Oily Fish: M D D 2.00, B D I 1.74, B D I I 1.74.

3. Respondents not meeting dietary guidelines (Counts and Percentages):

- D Q S: 92 percent of M D D, 94.7 percent of B D I, and 100 percent of B D I I do not meet guidelines.

- Fruit: 80 percent of M D D and 100 percent of both B D I and B D I I fail to meet guidelines.

- Vegetables: 74 percent of M D D, 89.5 percent of B D I, and 93.6 percent of B D I I fail to meet guidelines.

Navigate back to Table 1..

### Table 2. Long description

The table is organized into four main categories of eating behaviors. The columns represent three groups: M D D, B D I, and B D I I.

1. Binge eating behavior (counts and percentages):

- Little or no: M D D 20 (40), B D I 11 (57.9), B D I I 13 (41.9).

- Moderate: M D D 18 (36), B D I 4 (21.1), B D I I 10 (32.3).

- Severe: M D D 12 (24), B D I 4 (21.1), B D I I 8 (25.8).

2. Emotional eating (mean scores and standard deviations):

- Enjoyment of food: M D D 4.13 (.83), B D I 4.32 (.77), B D I I 4.29 (.95).

- Emotional overeating: M D D 2.87 (1.06), B D I 3.73 (.76), B D I I 3.09 (1.18).

- Emotional under-eating: M D D 3.07 (1.03), B D I 2.43 (.52), B D I I 2.53 (1.06).

- Food responsiveness: M D D 3.58 (.89), B D I 3.91 (.54), B D I I 3.53 (.87).

- Satiety responsiveness: M D D 2.62 (1.06), B D I 1.75 (.67), B D I I 2.18 (1.12).

3. Night eating behavior (counts and percentages):

- Normal: M D D 21 (42), B D I 10 (52.6), B D I I 15 (48.4).

- Mild: M D D 6 (12), B D I 2 (10.5), B D I I 6 (19.4).

- Moderate: M D D 14 (28), B D I 3 (15.8), B D I I 3 (9.7).

- Full syndrome: M D D 9 (18), B D I 4 (21.1), B D I I 7 (22.6).

4. Food addiction (counts and percentages):

- No food addiction: M D D 33 (66), B D I 17 (89.5), B D I I 26 (83.9).

- Moderate: M D D 3 (6), B D I 1 (5.3), B D I I 2 (6.5).

- Severe: M D D 14 (28), B D I 1 (5.3), B D I I 3 (9.7).

Navigate back to Table 2..

### Table 3. Long description

The table consists of 17 rows and 4 columns. The columns are Eating behavior score, P C 1, P C 2, and P C 3.

* B E S score: P C 1 is 0.65, P C 2 is minus 0.15, P C 3 is minus 0.61.

* D Q S score: P C 1 is minus 0.04, P C 2 is minus 0.09, P C 3 is 0.51.

* r N E D Q score: P C 1 is 0.73, P C 2 is minus 0.09, P C 3 is 0.00.

* Y F A S score: P C 1 is 0.68, P C 2 is minus 0.28, P C 3 is minus 0.37.

* Food fussiness: P C 1 is 0.03, P C 2 is 0.52, P C 3 is minus 0.24.

* Food responsiveness: P C 1 is minus 0.63, P C 2 is 0.62, P C 3 is minus 0.06.

* Enjoyment of food: P C 1 is minus 0.22, P C 2 is 0.76, P C 3 is 0.05.

* Emotional overeating: P C 1 is minus 0.34, P C 2 is 0.43, P C 3 is 0.08.

* Emotional undereating: P C 1 is minus 0.02, P C 2 is 0.75, P C 3 is 0.20.

* Slow necessitating: P C 1 is minus 0.04, P C 2 is 0.27, P C 3 is 0.81.

* Hunger: P C 1 is 0.81, P C 2 is 0.02, P C 3 is minus 0.03.

* Satiety responsiveness: P C 1 is minus 0.14, P C 2 is 0.83, P C 3 is 0.18.

* S S loadings: P C 1 is 2.64, P C 2 is 2.84, P C 3 is 1.57.

* Proportion variance: P C 1 is 0.22, P C 2 is 0.24, P C 3 is 0.13.

* Cumulative variance: P C 1 is 0.22, P C 2 is 0.46, P C 3 is 0.59.

* Proportion explained: P C 1 is 0.37, P C 2 is 0.40, P C 3 is 0.22.

* Cumulative proportion explained: P C 1 is 0.37, P C 2 is 0.78, P C 3 is 1.00.

Factor loadings with an absolute value greater than 0.30 are marked with a superscript a, and the highest loadings for each component are in boldface.

Navigate back to Table 3..

### Figure 1. Long description

The figure consists of four panels labeled A through D. A legend on the right identifies blue dots as Component 1, red dots as Component 2, and green dots as Component 3.

* Panel A is a scatter plot of Component 1 on the x-axis versus Component 2 on the y-axis. Data points are clustered around the origin with three overlapping ellipses. The blue ellipse is horizontally oriented, the red ellipse is vertically elongated and tilted left, and the green ellipse is smaller and more central.

* Panel B is a scatter plot of Component 2 on the x-axis versus Component 3 on the y-axis. The green ellipse is vertically elongated, while the red and blue ellipses are smaller and horizontally oriented, centered near the origin.

* Panel C is a scatter plot of Component 1 on the x-axis versus Component 3 on the y-axis. The blue ellipse is wide and horizontal, the green ellipse is tall and vertical, and the red ellipse is smaller and centrally located.

* Panel D is a 3D scatter plot showing the distribution of all three components simultaneously. The blue, red, and green dots are dispersed in a three-dimensional coordinate space with axes for Component 1, Component 2, and Component 3 ranging from approximately minus 2 to 2.

Navigate back to Figure 1..

### Table 4. Long description

The table contains six columns: Factor, d f (degrees of freedom), M S E (mean square error), F-value, ges (generalized eta squared), and p-value.

* Diagnosis: d f 2, 96; M S E 0.33; F-value 2.20; ges .009; p-value .116.

* Age: d f 4, 94; M S E 0.31; F-value 3.75 (significant at p < 0.01); ges .031; p-value .007.

* Gender: d f 2, 96; M S E 0.34; F-value 0.56; ges .002; p-value .574.

* Medication: d f 4, 94; M S E 0.32; F-value 2.71 (significant at p < 0.05); ges .022; p-value .035.

* B M I: d f 67, 31; M S E 0.29; F-value 1.26; ges .385; p-value .241.

* Cannabis use: d f 1, 97; M S E 0.34; F-value 0.17; ges less than .001; p-value .683.

* Alcohol use: d f 3, 95; M S E 0.34; F-value 0.56; ges .004; p-value .642.

* Exercise: d f 1, 97; M S E 0.34; F-value 1.45; ges .003; p-value .232.

* Smoking: d f 2, 96; M S E 0.34; F-value 0.88; ges .004; p-value .418.

Navigate back to Table 4..
