# Supplementary material for: Characterizing eating behavioral phenotypes in mood disorders: A cross-sectional investigation
Source: CNS Spectr. 2026 Sep 30;31(1):e42. doi: 10.1017/S1092852926101230 (PMC13732624; doi:10.1017/S1092852926101230)
Supplement: Supplementary file 1 [file S1092852926101230sup001.docx]

**SUPPLEMENTARY MATERIALS**

**Characterizing Eating Behavioral Phenotypes in Mood Disorders:**

**A Cross-Sectional Survey Investigation**

*Correlations Between Maladaptive Eating Behaviors*

**Figure A.** Correlational analysis of maladaptive eating behaviors in individuals with a mood disorder. The heatmap shows Pearson’s correlation coefficients with significant correlation values (p<0.01) indicated in boldface.

*Differences in the Prevalence of Maladaptive Eating Behaviors Between Diagnostic Groups*

| **Table A.** Diet Content and Quality | | | | | | |
| --- | --- | --- | --- | --- | --- | --- |
|  | | **Sum of Squares** | **df** | **Mean Square** | **F** | **Sig.** |
| **Diet Content (Fruit)** | **Between Groups** | **.356** | **2** | **.178** | **.572** | **.566** |
|  | **Within Groups** | **30.204** | **97** | **.311** |  |  |
|  | **Total** | **30.560** | **99** |  |  |  |
| **Diet Content (Vegetables)** | **Between Groups** | **.077** | **2** | **.038** | **.079** | **.924** |
|  | **Within Groups** | **47.363** | **97** | **.488** |  |  |
|  | **Total** | **47.440** | **99** |  |  |  |
| **Diet Content (Oily Fish)** | **Between Groups** | **1.690** | **2** | **.845** | **1.970** | **.145** |
|  | **Within Groups** | **41.620** | **97** | **.429** |  |  |
|  | **Total** | **43.310** | **99** |  |  |  |
| **Diet Content (Fat)** | **Between Groups** | **3.209** | **2** | **1.605** | **2.257** | **.110** |
|  | **Within Groups** | **68.981** | **97** | **.711** |  |  |
|  | **Total** | **72.190** | **99** |  |  |  |
| **Diet Content (NMES)** | **Between Groups** | **5.389** | **2** | **2.695** | **5.089** | **.008** |
|  | **Within Groups** | **51.361** | **97** | **.529** |  |  |
|  | **Total** | **56.750** | **99** |  |  |  |
| **Diet Quality Score** | **Between Groups** | **12.271** | **2** | **6.136** | **2.320** | **.104** |
|  | **Within Groups** | **256.479** | **97** | **2.644** |  |  |
|  | **Total** | **268.750** | **99** |  |  |  |

| **Table B.** Binge Eating Category | | | | | |
| --- | --- | --- | --- | --- | --- |
|  | **Sum of Squares** | **df** | **Mean Square** | **F** | **Sig.** |
| **Between Groups** | **.665** | **2** | **.333** | **.510** | **.602** |
| **Within Groups** | **63.335** | **97** | **.653** |  |  |
| **Total** | **64.000** | **99** |  |  |  |

| **Table C.** Adult Eating Behavior Questionnaire | | | | | | |
| --- | --- | --- | --- | --- | --- | --- |
|  | | **Sum of Squares** | **df** | **Mean Square** | **F** | **Sig.** |
| **Enjoyment of Food** | **Between Groups** | **.697** | **2** | **.348** | **.473** | **.625** |
|  | **Within Groups** | **71.432** | **97** | **.736** |  |  |
|  | **Total** | **72.129** | **99** |  |  |  |
| **Emotional Overeating** | **Between Groups** | **10.147** | **2** | **5.074** | **4.620** | **.012** |
|  | **Within Groups** | **106.533** | **97** | **1.098** |  |  |
|  | **Total** | **116.680** | **99** |  |  |  |
| **Emotional Under-eating** | **Between Groups** | **8.318** | **2** | **4.159** | **4.400** | **.015** |
|  | **Within Groups** | **90.749** | **96** | **.945** |  |  |
|  | **Total** | **99.067** | **98** |  |  |  |
| **Food Fussiness** | **Between Groups** | **.093** | **2** | **.047** | **.045** | **.956** |
|  | **Within Groups** | **100.711** | **97** | **1.038** |  |  |
|  | **Total** | **100.804** | **99** |  |  |  |
| **Food Responsiveness** | **Between Groups** | **1.869** | **2** | **.935** | **1.359** | **.262** |
|  | **Within Groups** | **66.706** | **97** | **.688** |  |  |
|  | **Total** | **68.575** | **99** |  |  |  |
| **Slowness in Eating** | **Between Groups** | **.451** | **2** | **.225** | **.222** | **.801** |
|  | **Within Groups** | **98.261** | **97** | **1.013** |  |  |
|  | **Total** | **98.712** | **99** |  |  |  |
| **Hunger** | **Between Groups** | **3.276** | **2** | **1.638** | **2.654** | **.075** |
|  | **Within Groups** | **59.862** | **97** | **.617** |  |  |
|  | **Total** | **63.138** | **99** |  |  |  |
| **Satiety Responsiveness** | **Between Groups** | **11.152** | **2** | **5.576** | **5.339** | **.006** |
|  | **Within Groups** | **101.300** | **97** | **1.044** |  |  |
|  | **Total** | **112.452** | **99** |  |  |  |

| **Table D.** Night Eating Diagnostic Score | | | | | |
| --- | --- | --- | --- | --- | --- |
|  | **Sum of Squares** | **df** | **Mean Square** | **F** | **Sig.** |
| **Between Groups** | **.687** | **2** | **.343** | **.275** | **.760** |
| **Within Groups** | **121.273** | **97** | **1.250** |  |  |
| **Total** | **121.960** | **99** |  |  |  |

| **Table E.** Yale Food Addiction Category | | | | | |
| --- | --- | --- | --- | --- | --- |
|  | **Sum of Squares** | **df** | **Mean Square** | **F** | **Sig.** |
| **Between Groups** | **4.118** | **2** | **2.059** | **3.551** | **.032** |
| **Within Groups** | **56.242** | **97** | **.580** |  |  |
| **Total** | **60.360** | **99** |  |  |  |

*Principal Component Analysis*

**Figure B.** Principal component analysis Scree plot.
